# Manipulation of the Symbiodiniaceae microbiome confers multigenerational impacts on symbioses and reproductive ecology of its *Exaiptasia diaphana* host

**DOI:** 10.1093/ismejo/wraf189

**Published:** 2025-09-02

**Authors:** Mark McCauley, Federica Montesanto, Samuel A Bedgood, Cody Miner, Keyla Plichon, Virginia M Weis, Sandra Loesgen

**Affiliations:** U.S. Geological Survey, Wetland and Aquatic Research Center, Gainesville, FL 32653, United States; Whitney Laboratory for Marine Bioscience, University of Florida, St. Augustine, FL 32080, United States; Department of Chemistry, Whitney Laboratory for Marine Bioscience, University of Florida, St. Augustine, FL 32080, United States; Whitney Laboratory for Marine Bioscience, University of Florida, St. Augustine, FL 32080, United States; Department of Chemistry, Whitney Laboratory for Marine Bioscience, University of Florida, St. Augustine, FL 32080, United States; Ecoscience Department, Aarhus University, Roskilde, DK-4000, Denmark; Department of Integrative Biology, Oregon State University, Corvallis, OR 97331, United States; Division of Natural Sciences, New College of Florida, Sarasota, FL 34243, United States; Whitney Laboratory for Marine Bioscience, University of Florida, St. Augustine, FL 32080, United States; Department of Biology, Whitney Laboratory for Marine Bioscience, University of Florida, St. Augustine, FL 32080, United States; Department of Integrative Biology, Oregon State University, Corvallis, OR 97331, United States; MSc MARRES, Université Côte d’Azur, Sophia Antipolis Campus, Nice, 06103, France; Department of Integrative Biology, Oregon State University, Corvallis, OR 97331, United States; Whitney Laboratory for Marine Bioscience, University of Florida, St. Augustine, FL 32080, United States; Department of Chemistry, Whitney Laboratory for Marine Bioscience, University of Florida, St. Augustine, FL 32080, United States

**Keywords:** Aiptasia, bacteria, fungi, pedal laceration, cnidarian

## Abstract

Symbiodiniaceae-associated microbiota strongly influence cnidarian symbioses. We systematically reduced the bacterial and fungal communities associated with Symbiodiniaceae to study potential effects on the cnidarian holobiont *Exaiptasia diaphana* (Aiptasia). Clonal anemones were inoculated with xenic *Breviolum minutum* (SSB01) and microbiome-manipulated cultures after antibacterial or antifungal treatment. The asexual reproduction of pedal laceration allowed for three generations of clonal aposymbiotic Aiptasia to be utilised in this study, from the initial adult generation (G0), to the first (G1), and second (G2) generation. We inoculated small and large G1 Aiptasia with SSB01 algae and monitored onset of symbiosis, rate of algal proliferation, and holobiont characteristics. Sequencing the 16S and 18S rRNA gene regions identified significant differences in the bacterial and fungal communities of the G0 and G1 generations, alongside differences between the size classes of small and large G1 anemones. The microbiome of larger G1 individuals was distinct to the smaller G1 anemones, suggesting a microbiome maturation process. Control *Breviolum minutum* cultures exhibited a significantly greater proliferation rate in large G1 anemones when compared to antibacterial or antifungal treated cultures, whereas the opposite trend was documented in the small G1 anemones. Although no differences were observed between algal photochemical parameters, or the growth and polyp activity of G1 juveniles, we observed a significant influence in the production of G2 clones between treatments. Overall, we provide strong ecological implications of manipulating Symbiodiniaceae microbiome, not for the algae themselves, but for the maturation of the host Aiptasia, as well as for the cnidarian holobiont over multiple generations.

## Introduction

Cnidarians (including corals, anemones, and jellyfish) are vital components of tropical marine ecosystems and have long been recognized for their complex relationships with a diverse range of microorganisms. These microorganisms include photosynthetic dinoflagellates in the family Symbiodiniaceae, alongside less studied communities of archaea, bacteria, fungi, microeukaryotes, and viruses (reviewed in [1–6]). Microbiota are not mere inhabitants but an integral component of the cnidarian *holobiont*, a concept that views the host and its associated microorganisms as a single ecological unit [7–11]. Microbial symbionts are key players in nutrient cycling, pathogen defense, and stress resilience, amongst other roles, and are important for the phenotype, health, adaptability, and evolutionary success of cnidarians [11–14].

Although the relationship between cnidarians and Symbiodiniaceae has been thoroughly investigated, and the cnidarian-microbiome (specifically bacteria) association is increasingly well-studied, little is known about Symbiodiniaceae-microbial relationships, or their potential influence on the fitness of the holobiont [15, 16]. Symbiodiniaceae strains maintain distinct bacterial communities [17, 18]. This is further correlated to cnidarian hosts, with highly heterogeneous Symbiodiniaceae and bacterial and fungal associations identified [19, 20]. However, many Symbiodiniaceae also host a few common bacterial genera, including *Labrenzia*, and *Mauricauda* [17, 18, 21]. The global distribution of these core bacteria suggests that they play an important role for the Symbiodiniaceae.

The sea anemone *Exaiptasia diaphana*, (hereafter referred to as Aiptasia), serves as a powerful model system for studying the symbiosis between cnidarians, Symbiodiniaceae, and their microbiota [22–24]. Given its close taxonomic placement to scleractinian corals, Aiptasia engages in symbiosis with similar microbial associates [3]. Aiptasia reproduces asexually through pedal laceration, a process in which mobile polyps (in our study these adult polyps are called G0) leave behind clusters of pedal disc cells that develop into new clonal polyps (in our study these clones are called the G1 generation) [25]. Further, in our study large G1 clones continued to asexually reproduce, creating a second generation of clones (the G2 generation). This reproductive strategy allows for the generation and maintenance of large clonal colonies in culture. Additionally, Aiptasia acquires endosymbionts from the environment with each new generation, facilitating experiments that manipulate symbionts independently [26].

Recent studies on the microbiome (specifically bacteriome) of Aiptasia have revealed significant insights into the relationships between bacterial communities and its symbiotic state with Symbiodiniaceae. The algal symbiotic state significantly influences the host microbiome and carrying capacity (abundance of bacteria hosted) [27, 28]. Additionally, key bacterial families remained stable, were enriched, or were only identified in Symbiodiniaceae hosting anemones [29, 30]. Significant differences in bacterial composition influenced by symbiotic state and host clonal line have also been found, with distinct bacterial taxa more abundant in either algal symbiont-free (aposymbiotic) or symbiotic anemones [31]. Key bacterial taxa are associated with four genotypes of Aiptasia from the Great Barrier Reef, noting a consistent core microbiome with some environmental influences [32]. Antibiotic impacts on the microbiome of Aiptasia resulted in significant reductions in microbial diversity, changes in community composition, and impacts to host development [33, 34]. Environmental variables including elevated temperature and reduced pH also decrease the bacterial community richness and evenness [35, 36]. Together, these studies underscore the complexity and importance of bacterial communities in Aiptasia, influenced by symbiotic state and environmental conditions.

Despite these advances, several aspects of Aiptasia microbial communities remain unexplored. We do not yet understand how microbial communities of Symbiodiniaceae influence the onset of symbiosis with the host, or how the algal-associated microbiome influences holobiont ecology and reproductive strategies, and the role of fungal communities in Aiptasia is still largely unknown. Fungi are known to play significant roles in other marine organisms, contributing to nutrient cycling, disease resistance, and symbiotic interactions, but their presence and function in Aiptasia or their algal symbionts have not been thoroughly investigated [37]. Additionally, it is not yet known how microbial and fungal communities may be influenced by the host developmental stage over time and over asexually-produced generations.

In this study, we aimed to investigate the influence of Symbiodiniaceae-associated bacterial and fungal communities on asexually-derived Aiptasia juveniles of different sizes, and across two generations (G1 and G2) produced by the process of pedal laceration from the initial G0 generation. Aiptasia individuals used in this study are of the H2 strain, a female clonal line originating from the Island of Hawaiʻi [38]. We conducted microbiome manipulations by creating three *Breviolum minutum* cultures (SSB01) and exposing the first to antibiotics, the second to antifungals, and the third remained as our untreated control. We then inoculated our clonal G1 population of two size classes (small and large) with these three algal cultures and kept one aposymbiotic condition. We followed the onset of symbiosis across these treatments, recording the proliferation of Symbiodinaceae in each individual, as well as observing the growth and behavior of G1 anemones, alongside measuring photosynthesis *in hospite*. In addition, we closely followed the production and growth of G2 anemones produced via pedal laceration by the large G1 anemones.

## Material and methods

### S‌SB01 algal culturing and antibiotic/fungal treatments

**Figure 1:**
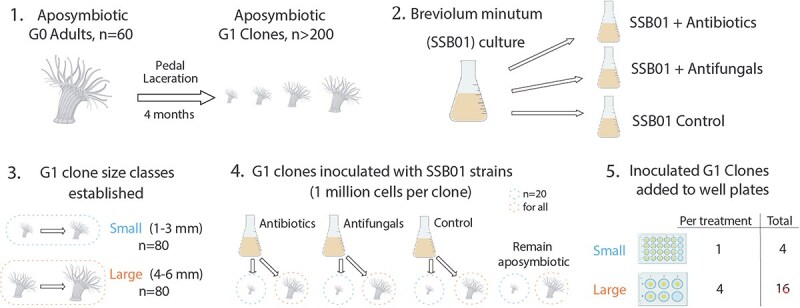
Schematic of experimental design; (A) Aiptasia G0 asexual reproduction through pedal laceration produced G1 individuals for this experiment; (B) Treatment of Symbiodiniaceae (*B. minutum*, SSB01) cultures with antibiotic or antifungal compounds to produce three distinct algal cultures; (C) Classifying size classes of G1 anemones into small (1–3 mm) and large (4–6 mm) based on their oral disc width; (D) Iinoculation of both small and large aposymbiotic anemones resulting in four experimental treatments; (F) Experimental containers for duration of the experiment allows for each individual to be tracked throughout the entire experiment (20 days for small anemones, 26 days for large anemones).

Clonal SSB01 (*B. minutum,* confirmed by ITS2 sequencing) liquid culture was provided by Oregon State University and grown in Guillard’s (F/2) Marine Water Enrichment Solution (Millipore-Sigma G0154) made in artificial seawater (hereafter ASW) (salinity before adding medium: ~32 ppt) at the Whitney Laboratory for Marine Bioscience, University of Florida. This culture was incubated without agitation at 25°C–26°C on a 12-h light/12-h dark cycle with an irradiance of ~40 μmol photons m^−2^ s^−1^ of photosynthetically active radiation provided by Nicrew White and Blue LEDs. At a density of ~9 million cells (cells enumerated with replicate hemocytometer counts), this culture was equally split into three distinct cultures (Fig. 1). One of these cultures (“Antibacterial treatment”) was treated with 100 μg/ml penicillin–streptomycin (100 μg/ml) and 10 mg/ml kanamycin for 1 month to reduce the bacterial component of the Symbiodiniaceae-associated microbiome [39]. The “Antifungal treatment” culture was similarly treated for 1 month with 125 μg/ml amphotericin B and 2.5 mg/ml nystatin to reduce fungal associates. The control SSB01 culture remained untreated.

### H2 Aiptasia inoculation and experimental design

The clonal Aiptasia H2 strain was used in this study. Anemones were made fully aposymbiotic (cleared of their Symbiodiniaceae) through menthol bleaching [40] at Oregon State University and then shipped to the Whitney Lab. Aposymbiotic status was confirmed upon arrival with epifluorescence microscopy (detailed below). Anemones were maintained in filtered (0.2 μm) ASW in opaque polycarbonate containers at 25°C–27°C on a 12-h light/12-h dark cycle with feeding with freshly hatched *Artemia salina* three times a week and a water change ~8 h after feeding.

Approximately 60 aposymbiotic adults (G0 generation) produced >200 aposymbiotic pedal lacerates over a 4-month time period (G1 generation). G1 juveniles were classified according to the width of their oral disc as either being small (1–3 mm, *n* = 80) or large (4–6 mm *n* = 80) (Fig. 1). Prior to inoculation with one of the three algal treatments, all G1 juveniles were confirmed to be aposymbiotic by epifluorescence. Twenty small and large G1 anemones were transferred into transparent containers and simultaneously inoculated with ~1 × 10^6^ cells/ml per individual anemone for each of the three different experimental treatments of the algal cultures (“Antibiotic,” “Antifungal,” and “Control”), administered with macerated *Artemia* (Fig. 1). An equal number of small and large anemones were left uninoculated but also transferred into transparent containers and similarly fed macerated *Artemia*. After 48 h, anemones in each treatment underwent a water change and were gently rinsed with ASW to remove any remaining Symbiodiniaceae. Inoculation success for each individual was confirmed by epifluorescence microscopy before being transferred to 6- (large anemones) or 24-well polypropylene plates (small anemones) (Fig. 1).

A single Aiptasia individual was maintained in each well to allow for precise tracking of anemones throughout the experiment. Large G1 anemones continued to produce clonal G2 anemones through pedal laceration, which were carefully removed when identified and placed in a separate container and monitored until the end of the experiment. The experiment continued until accurate imaging of algal density became challenging due to overlapping epifluorescence signals, which given the reduced volume within the smaller anemones occurred 20 days post inoculation and 26 days post inoculation for the larger size class. At these timepoints (day 20 for small; day 26 for large), G1 anemones were sacrificed for DNA extraction.

### Epifluorescence imaging for algal quantification

Symbiodiniaceae density was visualized for all G1 anemones with epifluorescence imaging, and each individual (*n* = 160) was tracked across the entire experiment. We used a Zeiss Axioskop 2 FS fluorescence microscope to capture algal autofluorescence (Zeiss filter set, excitation: 546/12 nm, dichroic: 580 nm, emission: long-pass 590 nm) using a Basler camera (Model: acA2240 – 35um). Imaging and quantification followed the protocol of [41], with calculations in ImageJ. Briefly, z-stack videos (100 frames) were recorded for each individual and then merged into a single image. This image was converted from color into a greyscale, allowing for the program to identify and enumerate algal densities (Supplementary Fig. 1). Whole tentacles (triplicate, of similar sizes) were imaged and tracked throughout the experiment. Rates of algal proliferation were calculated for each individual G1 anemone, using the quantifiable algal counts acquired every 2 days until the experiment was ended.

### Photochemical efficiency of Symbiodiniaceae

Photosynthetic parameters were determined using Pulse Amplitude Modulation fluorescence analyzer MINI-PAM-II (Walz GmbH, Germany) with maximum quantum yield (Fv/Fm) measured three times a day (6 a.m., 6 p.m., and 9 p.m.). These times corresponded to the end of the “night” cycle, the end of the “day” cycle, and again 3 h later to record potential immediate photosystem recovery. Before inoculation, all aposymbiotic Aiptasia were measured *in vivo* at a constant distance (~10 mm) to ensure no light-adapted quantum yield was recorded, then light-adapted quantum yield was measured for small and large Aiptasia daily throughout the experiment. Additionally, at the start and at the end of the experiment, rapid light curves were conducted in triplicate on each of the three treatment cultures (20 s intervals), following the protocol of [42].

### Growth, reproduction, and behavior of Aiptasia individuals

The growth of pedal disc diameter was measured for both small and large anemones throughout the experiment with images taken from the bottom of the well plate at the same time every 2 days. Additionally, the production of pedal lacerates (G2 generation) from G1 offspring was recorded daily with the identified G2 pedal lacerates isolated. Given their small size, G2s were broadly categorized by their color (translucent, pale yellow, light beige, and dark brown), to infer the relative abundances of Symbiodiniaceae, at the end of the experiment [43].

Observations were made every other day to assess the behavior (polyp activity) of the anemones. For each individual, the proportion of tentacles that were expanded vs retracted tentacles were recorded. These observations were made repeatedly at the same time of day, and after the anemones had not been fed or been handled for a period of 6 h.

### Amplicon gene region sequencing (16S rRNA, 18S rRNA, and ITS2)

A total of 64 anemones were individually homogenized using a mortar and pestle and DNA extraction was performed using the Qiagen DNeasy PowerSoil kit (Qiagen, CA, USA), according to Quick Start protocol (May 2019), with two modifications (vortex time was doubled from 10 to 20 min, with a final volume of elution 25 *μl)*. Additionally, samples from each of the three treatment algal cultures, and six blank DNA extractions were included in order to test for potential bacterial contamination of the DNA extraction kit and/or reagents and primers. The quantity and quality of extracted DNA were measured using a Qubit 3 fluorometer (FisherSci). The 16S rRNA gene (V4 hypervariable region) was amplified to characterize the bacterial community using the primer set 515F (Parada) (5′-GTGYCAGCMGCCGCGGTAA-3′) and 806R (Appril) (5′-GGACTACNVGGGTWTCTAAT-3′) [44, 45]. In addition, a region of ~348 bp of the 18S rRNA gene (V7-V8 hypervariable region) was amplified to characterize the fungal community with the fungi specific primer set nu-SSU-1333-5 (5′-CGATAACGAACGAGACCT-3′) and nu-SSU-1647-3 (5′-ANCCATTCAATCGGTANT-3′) [46]. Finally, algal endosymbiont identity was confirmed using a region of estimated amplification size ~234–266 bp length of the ITS2 gene with the primer set SYM_VAR_5.8S2 (5′-CAGCTTCTGGACGTTGYGTTGG-3′) and SYM_VAR_REV (5′-CGGGTTCWCTTGTYTGACTTCATGC-3′) [47].

Amplicon quantities were confirmed with a Qubit 3 fluorometer (FisherSci), and quality checked on a spectrophotometer (BioTek Take 3 Plate, Agilent) by the 260/280 nm ratio. DNA concentrations were then normalized and sent to Genewiz (Azenta) for sequencing on a MiSeq platform (Illumina; 2 × 250 bp). When significant quantities of DNA were extracted from anemones, all three gene regions were amplified. However, in most cases given the small size of the anemones, there was often enough DNA for the amplification of only one or two gene regions. In these cases, 16S rRNA gene region sequencing was prioritized, followed by 18S rRNA gene region sequencing, and then ITS2 gene region sequencing.

### Microbiome analysis

Sequences were filtered and trimmed using the DADA2 R-package (v1.18–1.22 [48]) in RStudio (2021.09.0351 [48]), allowing for the calculation of a distinct error rate. Sequences were processed according to the DADA2 SOP (https://benjjneb.github.io/dada2/tutorial.html). All sequences were processed with the filtering qualifications of (maxN = 0, maxEE = 2,2), before being merged, having chimeras removed, and taxonomically classified with a naïve Bayesian method using DADA2. A total of 43,608 16S rRNA gene region amplicon sequence variants (ASVs) were identified across 73 samples, and 2,724 18S rRNA gene region ASVs were identified across 50 samples.

Taxonomic annotation of sequences was performed with the Silva database (v. 138.2 SSU [49]), and PR2 (v. 5.0.1 [50]). To increase the detection rate of 18S rRNA gene sequences the database was adapted by inserting partial and full-length sequences from the NCBI fungi dataset. 16S rRNA gene ASVs that were not classified to the Kingdom “Bacteria,” or were identified as the Order “Chloroplast,” or the Family “Chloroplast” were removed. 18S rRNA gene ASVs that were not identified as the Kingdom “Fungi,” were similarly removed. Further, sequences identified to Kingdom Fungi were examined with the NCBI BLAST function for increased taxonomic resolution. Using the phyloseq R-package (v1.38.0 [52]), the 16S rRNA gene dataset was rarified to 248,946 sequences, with the removal of two samples, and the 18S rRNA gene dataset was rarified to 52,250 sequences, with the removal of two samples. Given the reduced and uneven distribution of samples that the 18S rRNA gene region was sequenced from, and the significantly higher rate of sequences that were unassigned to the Kingdom Fungi, we have constrained our fungal statistical analyses to aposymbiotic Aiptasia samples and only highlight patterns in symbiotic anemones. ITS2 sequences confirmed the identity of our algal endosymbionts as *Breviolum minutum*. Sequences were merged with their metadata and exported using the phyloseq R-package (v1.38.0 [49]) for further analysis.

### Statistical analysis

Repeated Measures ANOVAs were conducted on algal abundances once assumptions (normality, presence of outliers, homogeneity of variance, and homogeneity of sphericity) had been checked and confirmed. A Bonferonni’s *post hoc* test determined differences between treatments with the rstatix R-package (v0.7.2 [51]).

For microbial analyses, Inverse-Simpson, Shannon, Fisher’s, and Observed diversity metrics were tested with a linear mixed-effects model [52–54] using the lme4 R-package (v1.1–27.1 [55]) and plotted using the ggplot2 R-package (v3.3.5107 [56]). Bray–Curtis B-diversity, Weighted Unifrac distances were calculated using a PERMANOVA (999 permutations), alongside tests for dispersion, with the vegan R-package (v2.5–7106 [57]). Samples were hierarchically clustered (complete linkage) using dissimilarities and plotted with the stats R-package (v4.1.288 [58]) and the phyloseq R-package (v3.6.2 [49]). The differential abundances were calculated with DESeq (v2.12 [59]), and the heatmaps were produced with the ampvis2 R-package (v.2.8.9 [60]). The heat trees were produced with the metacoder R-package (v0.3.7 [61]), the Upset plots were created with the UpSetR R-package (v1.4.0 [62]), and the Venn diagrams were plotted with the ggplot2 R-package (v3.3.5107 [56]).

## Results

### Microbial community comparison between SSB01 treatments

**Figure 2:**
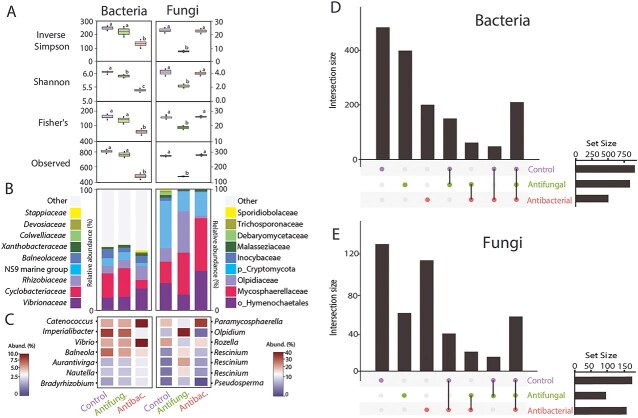
Bacterial (16S rRNA gene V4 region) and fungal (18S rRNA gene V7-V8 region) community comparison between three SSB01 experimental treatment cultures; (A) Significant microbiome manipulation observed in bacterial and fungal communities, illustrated across multiple alpha diversity indices for richness, abundance, and evenness across anemones size class; (B) Top 10 most abundant bacterial and fungal families across anemones size class; (C) Heatmap displaying the top seven genera of bacteria and fungi that were most significantly different between the culture treatments; Unique and shared (D) bacteria, and (E) fungi, identified across the three algal cultures.

Manipulation of Symbiodiniaceae microbiomes were successful with significant reductions in the diversity of the bacterial and fungal communities (Fig. 2A). Short-term exposure to antibacterials and antifungals did not render these algae axenic, but they did result in significant changes (ANOVA, *P* < .01, for all) to the structure of bacterial and fungal communities, respectively (Fig. 2A). Increases in the relative abundance of the bacterial family Vibrionaceae and genera *Catenococcus* and *Vibrio* were observed with antibacterial exposure, with concurrent reductions in the relative abundance of the family *Cyclobacteriaceae* and the genera *Imperialibacter* and *Balneola* (Fig. 2B and C). Antifungal exposure increased the relative abundance of the fungal genera *Olpidium* and *Rescinium* associated with the Symbiodinaceae alongside decreasing relative abundances of the family Cryptomycota and the genera *Paramycosphaerella* and *Rozella* (Fig. 2B and C). Although the control and antifungal cultures hosted many unique ASVs, they also hosted more shared ASVs than either culture did with the antibiotic treated culture (Fig. 2D). This pattern was also observed with the antifungal treated culture, whereby it shared few ASVs with both the control and antibiotic treated cultures (Fig. 2E). No significant differences were detected in the photosynthetic efficiencies of the three algal culture treatments, determined by conducting rapid light curves at both the start or the end of the experiment (ANOVA, *P* > .05; Supplementary Fig. 2).

### Microbial community comparison between aposymbiotic G0 anemones vs aposymbiotic G1 anemones

Significant community differences (ANOVA, *P* < .01 for all) were observed between aposymbiotic G0 anemones and their clonal aposymbiotic G1 anemones (Supplementary Fig. 3A). Small G1 anemones consistently hosted less diverse bacterial communities (Supplementary Fig. 3A). Large G1 anemones more closely resembled the bacterial community of the G0 adult (Supplementary Fig. 3A). There was less distinction in the fungal communities, with the large G1 anemones exhibiting a greater diversity when compared to the small G1 anemones (Supplementary Fig. 3A). However, when examining the identity of the microbiota, the large G1 size class more closely resembled the adult G0 than the small G1 in both bacterial and fungal communities (Supplementary Fig. 3B and C). This was similarly observed across Bray–Curtis distance analyses and with the relative abundance of shared ASVs between the adult G0 and larger size class (Supplementary Fig. 3D–G). Lower relative abundances of the bacterial genus *Blastopirellula* and higher relative abundances of the fungal genus *Basidiobolus* were identified in the G0 generation when compared to G1 (Supplementary Fig. 3C).

### Microbial community comparison between aposymbiotic G1 anemone size classes

**Figure 3:**
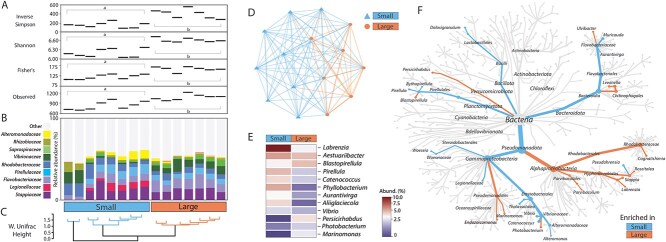
Bacterial (16S rRNA gene V4 region) community comparison between small and large G1 aposymbiotic anemones. (A) Significant differences observed across multiple alpha diversity indices, including richness, abundance, and evenness across the anemone size classes. (B) Top 10 most abundant bacterial families across the size classes. (C) Weighted UniFrac distance clustering per individual in the small and large size classes. (D) Bray–Curtis (beta diversity) dissimilarity network. (E) Heatmap displaying the top 12 genera of bacteria found to be most significantly different between the size classes. (F) Heat tree displaying the relatedness of bacteria found to be significantly enriched in each size class.

**Figure 4:**
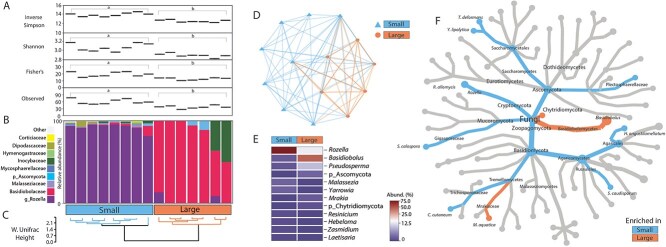
Fungal (18S rRNA gene V7-V8 region) community comparison between small and large aposymbiotic G1 anemones; (A) Significant differences observed across multiple alpha diversity indices, including richness, abundance, and evenness across the anemone size classes; (B) Top 10 most abundant fungal families across the size classes; (C) Weighted UniFrac distance clustering per individual in the small and large size classes; (D) Bray–Curtis (beta diversity) dissimilarity network; (E) Heatmap displaying the top 12 genera of bacteria found to be most significantly different between the size classes; (F) Heat tree displaying the relatedness of fungi found to be significantly enriched in each size class.

Bacterial community observed abundance (Observed diversity), evenness (Shannon), and diversity (Inverse Simpson, Fisher’s), were significantly lower in small aposymbiotic anemones compared to larger ones (ANOVA, *P* < .01, for all), whereas the opposite was observed for fungal communities (ANOVA *P* > .05, for all) (Figs 3A and 4A). Across anemone size classes, the bacterial families *Legionellaceae*, *Vibrionaceae*, and *Alteromonadaceae* and the fungal family *Basidiobolaceae* and the genus *Rozella* were most varied by relative abundance (Figs 3B and 4B), driving the significant microbial differences between small and large G1 juveniles (PERMANOVA, *P* < .001, for all) (Figs 3C and 4C). Significant bacterial and fungal clustering within size classes were detected with both Weighted UniFrac and Bray–Curtis distance analyses (PERMANOVA, *P* < .01, for all) (Fig. 3C and D;  Fig.  4C and D).

**Figure 5:**
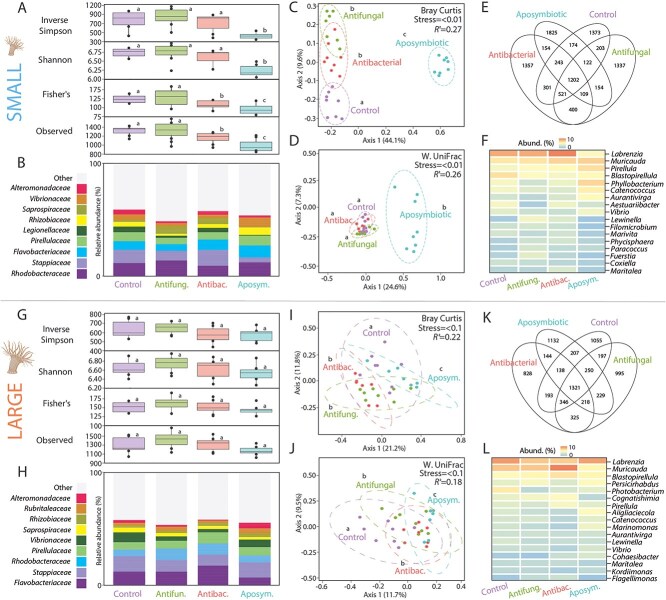
Bacterial (16S rRNA gene V4 region) community comparison between G1 anemones across four experimental treatments; (A, F) Visualize data from small anemones; (G, L) Visualize data from large anemones; (A, G) Significant differences observed across multiple alpha diversity indices for richness, abundance, and evenness across the four experimental treatments; (B, H) Top 10 most abundant bacterial families across the four experimental treatments; (C, I) Significant differences detected across Bray–Curtis dissimilarity (community composition) and (D, J) Weighted UniFrac dissimilarity (community relatedness) metrics; (E, K) Venn diagram of overlapping bacteria across the four experimental treatments; (F, L) Heatmap displaying the most significantly variable bacterial genera across the four experimental treatments.

The bacterial genera more abundant in both small and large individuals were *Rhodobacteraceae*, *Pirellulaceae*, *Flavobacteriaceae*, followed by *Saprospiraceae*, *Stappiaceae*, *Rhizobiaceae*, and *Vibrionaceae* (Fig. 5B and H). For the fungal communities, multiple genera were significantly varied between size classes, but the greatest variability came from the genera *Rozella*, *Basidiobolus*, and *Pseudosperma* (Fig. 4E and F).

### Microbial community comparison between experimentally inoculated G1 anemones

Small G1 aposymbiotic anemones had significantly lower richness and diversity of bacteria than small symbiotic anemones (ANOVA, *P* < .02) (Fig. 5A). Although the smaller anemones within the antibacterial treatment hosted a less rich community than the control and antifungal treatments (ANOVA, *P* < .05) (Fig. 5A), this trend was not observed with larger anemones, with no significant differences in alpha metrics detected across treatments (ANOVA, *P* > .05) (Fig. 5G). The four most common bacterial families (*Rhodobacteraceae*, *Stappiaceae*, *Flavobacteriaceae*, and *Pirellulaceae*) represented between 40% and 50% of the bacteriome in both sizes of juveniles and across all treatments (Fig. 5B and H).

Bray–Curtis and Weighted UniFrac dissimilarity analysis revealed that the presence of Symbiodiniaceae drives strong and significant differences between bacterial and fungal communities of both small and large G1 anemones (PERMANOVA, *P* < .01, for all) (Fig. 5C, D, I, and J). This was further evident when identifying bacterial ASVs retained in the Aiptasia holobiont from the initial SSB01 inoculation through the onset of symbioses, with 53.6% and 32.8% of the cultured SSB01 microbiome still present in the small and large G1 anemones respectively at the end of the experiment. Antibiotic and antifungal treatments also resulted in significant differences when compared to the control in both size classes (PERMANOVA, *P* < .01, for all) (Fig. 5C, D, I, and J). In small G1s, we observed more overall microbiome similarity, within and between treatments, when compared to larger anemones (Fig. 5C, D, I, and J). Although treatments across both size classes had similar ratios of bacteria shared across treatments (Fig. 5E and K; Supplementary Fig. 4A and B), a more abundant core community (bacteria identified in 80% of anemones at higher than 0.1% relative abundance) was detected in the large size class (46 ASVs), in comparison to the smaller size class (4 ASVs) (Supplementary Fig. 5).

Generally, all G1 anemones shared several microbial ASVs, with each one also hosting unique microbiota (Fig. 5E and K). *Labrenzia* was the most variable bacterial genus across both size classes, with the highest relative abundance recorded in the small antibacterial anemones and the lowest relative abundances in the aposymbiotic G1 anemones, but the opposite was observed in the large anemones (Fig. 5F and L). The genus *Muricauda* was the second most variable genus across both size classes with large and small anemones similarly revealing opposing patterns. In small anemones, the highest relative abundance was present in aposymbiotic individuals, whereas in the large anemones aposymbiotic individuals hosted the lowest relative abundance. Other differences were present across size classes, with both *Pirellula* and *Blastopirellula* more enriched in aposymbiotic small G1s when compared to antibacterial treated individuals, whereas in large anemones both genera were more enriched in antibacterial treated individuals in comparison to aposymbiotic anemones (Fig. 5F and L). Finally, the genera *Phyllobacterium* was relatively more abundant in small aposymbiotic G1’s, compared to all symbiotic treatments (Fig. 5F), with *Aliiglaciecola* and *Marinomonas* more abundant in large aposymbiotic G1’s (Fig. 5L).

The G1 anemones inoculated with the antifungal treated culture hosted a reduced fungal community, a pattern observed in both alpha and beta diversity indices (Supplementary Fig. 6A and D). Changes in the most relatively abundant fungal families and genera were apparent, with distinct reductions in the genera *Rozella* and *Basidiobolus* in the small and large anemones concurrent with increases in *Basidiobolus* and *Pseudosperma* in small and large anemones, respectively (Supplementary Fig. 6B and C). Fungal communities within these anemones were also more dissimilar to both those in the anemones inoculated with the control and antibacterial cultures (of both size classes) (Supplementary Fig. 6D and E). Although all anemones hosted highly unique fungal communities, smaller anemones shared more fungal ASVs across treatments than the larger size class (Supplementary Fig. 6F and G).

### Onset of Symbiodiniaceae symbioses in G1 anemones

**Figure 6:**
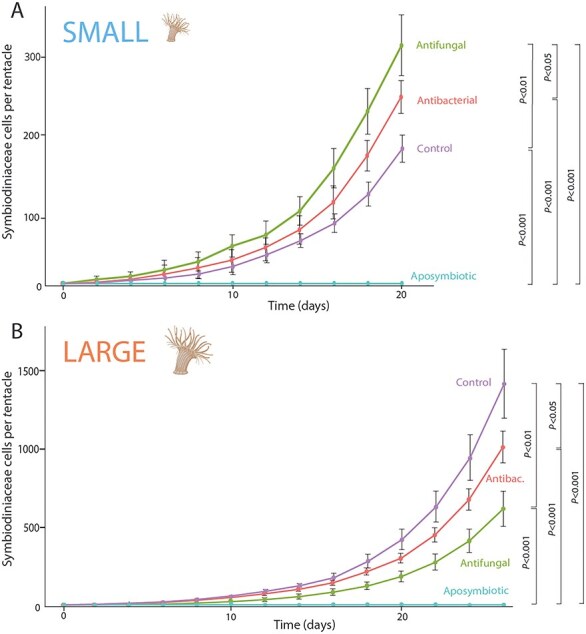
Quantification of *B. minutum* colonization of small and large G1 anemones; Symbiodiniaceae abundances (average of 20 anemones per datum) enumerated every 2 days across all four treatments in (A) small and (B) large anemones.

All anemones were checked prior to inoculation and 48 h post inoculation to determine if exposure to the Symbiodiniaceae cultures were successful. Significantly similar rates of uptake were observed across all treatments and across both size classes at the start of the experiment (Supplementary Fig. 7). The rate of algal proliferation was similar across size classes, with an average of 47.6 and 42.3 new Symbiodiniaceae cells per day (20 days post inoculation) in the small and large anemones respectively (Supplementary Fig. 7). However, the proliferation rate of Symbiodiniaceae populations were significantly different across all treatments within each size class (Fig. 6A and B). In small G1 anemones, control *B. minutum* algae had the slowest population growth, with antifungal (Repeated Measures ANOVA, *P* < .01) and antibacterial (Repeated Measures ANOVA, *P* < .05) treatments significantly outpacing untreated algae. However, the opposite was observed in the large G1 anemones, with significantly more *B. minutum* present in individuals infected from control cultures when compared to either antibacterial (Repeated Measures ANOVA, *P* < .05) or antifungal (Repeated Measures ANOVA, *P* < .01) treatments (Fig. 6A and B). In both size classes, no Symbiodiniaceae were detected in any of the aposymbiotic individuals throughout the experiment.

### Symbiodiniaceae photosynthetic performance, growth, and behavior of G1 anemones

No significant treatment effects on photosynthetic efficiency were detected throughout the experiment (ANOVA, *P* > .05) (Supplementary Fig. 8A). G1 growth was significantly slower in aposymbiotic compared to symbiotic anemones, regardless of size (ANOVA, *P* < .05) (Supplementary Fig. 8B). No treatment significantly influenced anemone behavior, although smaller individuals retracted their tentacles more often than larger ones (ANOVA, *P* > .05, for all) (Supplementary Fig. 8C).

### Production of G2 anemones, their size, and inferred algal densities

Large aposymbiotic G1 anemones produced significantly more G2 pedal lacerates than all other treatments (ANOVA, *P* < .05, for all) (Supplementary Fig. 9A). Large G1 anemones inoculated with Symbiodiniaceae treated with antibacterial and antifungal agents produced more lacerates than those inoculated with the control culture (ANOVA, *P* < .01 for all) (Supplementary Fig. 9A). This trend was observed from the onset of the symbiosis. At the end of the experiment, G2 juveniles produced by G1 anemones inoculated with antibacterial, and antifungal-treated algal culture, were significantly larger than those produced by control G1 lacerates inoculated with the control culture (ANOVA, *P* < .05 for all) (Supplementary Fig. 9B). G2 juveniles produced by large G1 aposymbiotic anemones were also larger, although we can’t identify if this was a result of algal treatment or if G2 development duration was longer for clones that were produced earlier than others (Supplementary Fig. 9B). Additionally, G2 anemones colonized with the antibacterial- and antifungal-treated algae qualitatively appeared darker than those of control anemones, likely reflecting a higher density of Symbiodiniaceae (Supplementary Fig. 9B).

## Discussion

### Maturation of the host microbiome through time

Given the Aiptasia model system’s ability to advance our understanding of cnidarian symbioses, one fundamental question to explore is how its microbiome is acquired and potentially matures through host development. We had hypothesized that large G1 individuals would have the same microbiome as small G1 individuals, which in turn would resemble the G0 generation, as they are the same clonal organism. However, we found significant changes in both the composition and diversity of both fungal and bacterial communities between G0 and G1 generations, and between small vs large aposymbiotic G1 juveniles (Figs 2–4; Supplementary Fig. 3). This may be related to a change in host selective pressures that “refines” the microbiome over time, for example by changing the relative abundance of dominant fungi or building a broader bacterial community [63]. This maturation is supported by the tighter microbial compositions in the smaller G1s between treatments compared to broader compositions in larger G1s (Fig. 5C, D, I, and J, Supplementary Fig. 3D and E). In addition, there was a greater distinction between the microbial communities of the treatments in the small G1s suggesting that the introduction of Symbiodiniaceae-associated microbiota had a greater influence on shaping the microbiome of the smaller anemone holobiont than in the larger G1s.

Bacterial communities differed significantly between G1 size classes (Fig. 3;  Fig.  4A, B, and D), with *Alphaproteobacteria* most enriched in larger anemones, and *Firmicutes* and *Gammaproteobacteria* often enriched in smaller anemones (Fig. 3F). The most significantly variable bacteria between size classes were *Legionellaceae*, *Pirellula, Phyllobacterium*, and *Marinomonas*. *Phyllobacterium* are typically associated with plant roots and known for their nitrogen-fixing capabilities (Fig. 3B, E, and F). These taxa could play a role in nitrogen cycling, providing essential nutrients to the anemone and algae, thereby supporting the symbiotic relationship [64]. *Marinomonas*, adept at degrading various organic compounds [65], may aid in the breakdown of organic matter, contributing to the nutrient pool available to the host and symbionts. Members of the *Legionellaceae* family, known for their ability to thrive in aquatic environments, often act as intracellular pathogens and may modulate the host immune response, thereby influencing the health and stability of the symbiotic relationship. However, there is the possibility that *Legionella* is a transient microbiont as was found in corals, and may be associated with human activities [66]. The prediction of these roles rely on studies from non-Aiptasia hosts and they should be examined within Aiptasia to determine their functional contribution to the holobiont.

In small G1 anemones, the enrichment of *Firmicutes* and *Gammaproteobacteria* is indicative of a microbial community specialized for rapid growth and nutrient uptake in response to the dynamic environment of smaller individuals, regenerating tissue [67]. For instance, the presence of *Marinomonas*, known for degrading organic compounds [68], may be particularly beneficial in developing small G1s by aiding in the rapid recycling of nutrients necessary for tissue regeneration. However, as the juveniles grow larger, the functional contributions of these bacteria may diminish, potentially due to changes in the nutrient composition and environmental conditions of larger animals. There were two differences in the experimental design of the size classes to address, small anemones were held in 24 well plates and sampled 20 days post inoculation, and large anemones held in 6 well plates and sampled 26 days post inoculation. Although both of these factors may have contributed to some of the microbial differences observed in the study, it is unlikely that they would have significantly influenced the results given the extent of variability observed in both the fungal and bacterial assemblages across multiple community indices.

Even though algal and bacterial members of cnidarian holobionts are commonly studied, very few studies also examine the fungal communities, and these have remained unexplored in Aiptasia. We identified distinct differences in the fungi associated with the G1 anemones, specifically *Rozella* and *Basidiobolus* (Fig. 4; Supplementary Fig. 6). *Rozella* species are known for being obligate intracellular parasites of other fungi and algae [69]. Their presence may indicate interactions with other microbial components within the anemone holobiont, potentially affecting the overall microbial balance [69]. *Basidiobolus* which belongs to the Order Entomophthorales, is primarily known for its saprophytic lifestyle, decomposing organic matter, and contributing to nutrient cycling [70]. In small G1s, these fungi could contribute to the breakdown and recycling of organic matter, facilitating rapid growth and tissue regeneration. However, as the juveniles grow larger, the functional contributions of these fungi could change, due to the stabilization of the microbial community and changes in nutrient dynamics.

Overall, there were microbial distinctions between the anemones size classes, but the majority of the relatively highly abundant bacteria identified in this study have been previously recognized as commonly associated with Aiptasia. Primarily, the families *Alteromonodacaeae*, *Flavobacteriaceae*, *Pirellulaceae*, *Rhodobacteraceae*, and *Vibrionaceae*, that contribute ~30% of the relative abundance in our anemones (Fig. 3B;  Fig.  5B and H; Supplementary Fig. 3B) were previously identified as being significant contributors to Aiptasia bacterial communities [27, 28, 30, 36, 71–73]. We found *Legionellaceae* significantly enriched in smaller G1 anemones, but did not find any previous evidence for this association. It is possible that they are predominantly present in smaller anemones, as all previous publications have either used only larger anemones or anemone size information was not included. Essentially, the microbial community may be contingent on the developmental stage of Aiptasia. Anemone size and maturation are important factors for consideration in future studies.

### Symbiodiniaceae treatments influence microbiomes of small anemones more than large ones

Treatment of Symbiodiniaceae cultures prior (Fig. 2) to their infection with both small and large aposymbiotic anemones significantly influenced their respective microbiomes at the end of the experiment (Fig. 5). This underscores the importance of the Symbiodiniaceae microbiome in contributing to the Aiptasia holobiont, in this instance shaping its microbial community. The strength of this influence was correlated to the size of the infected anemone, with the microbial communities of smaller G1 anemones more influenced than the large (Fig. 5). Overall, the strongest variable was the presence of Symbiodiniaceae, with aposymbiotic anemones exhibiting more dissimilar microbiomes even when compared to the antibacterial and antifungal treatments. Although the treatment differences were highly significant in the small G1 anemones (Fig. 5C and D), they were less obvious in the larger anemones (Fig. 5I and J), with samples overlapping to a greater degree, further providing evidence for microbiome maturation in Aiptasia.

There were shifts with the various treatments in the relative abundance of microbiota commonly identified as being beneficial to cnidarians, including *Labrenzia*, and *Muricauda. Labrenzia* was significantly enriched in symbiotic G1s, especially with antibacterial treatment. It is known to provide substantial metabolic benefits from partnerships with Symbiodiniaceae [39]. *Labrenzia* is one of the conserved bacterial genera maintained across global Symbiodiniaceae cultures, and produces dimethylsulfoniopropionate [74], which is important for stress tolerance [75, 76]. Similarly, *Muricauda* plays a role in nutrient cycling, inter-kingdom recognition, and interactions, and also provides stress protection by producing antioxidants such as zeaxanthin, which improve photosynthetic efficiency and neutralize reactive oxygen species during temperature stress [39].

*Vibrio* species, found in higher relative abundance in aposymbiotic anemones of both size classes, are well-known marine bacteria that can act as opportunistic symbionts or pathogens [77]; their increased presence in the aposymbiotic state may indicate a disrupted microbiome, potentially leading to stress or disease in the host [78, 79]. Although it is possible that the abundance of *Vibrio*, or *Muricauda*, may have been influenced through the feeding of *Artemia*, who can carry *Vibrio* species given their ability to degrade chitin [80, 81], both were present in the algal cultures prior to the introduction of *Artemia* to the system (Fig. 2B). In symbiotic cnidarians, however, *Vibrio* spp. seem to be members of the apparently healthy microbial community, with no known links to disease [79]; some contribute to nitrogen fixation [81–84] and defense of the coral against pathogens [78, 85]. Moreover *Vibrio* species have also been reported to induce metamorphosis in invertebrate larvae (e.g. *Cassiopea andromeda* [86])*.*

Shifts in the microbiota were often confounded by size class, including in the enrichment of *Pirellula*, and *Blastopirellula*. In our small G1 anemones, *Pirellula* was significantly enriched in the aposymbiotic treatment and yet was highest in symbiotic antifungal treatment of large anemones, whereas *Blasotopirellula* was significantly enriched in small G1 control anemones and in the antibacterial treatment for the large G1 anemones (Fig. 5F and L). Both genera belong to Phylum *Planctomycetota*, which is recognized for its ability to degrade complex organic matter and may contribute to nutrient cycling and the breakdown of organic material within the Aiptasia holobiont [87]. *Pirellula* spp., are often found in nutrient-poor oligotrophic environments and are capable of degrading a variety of polysaccharides due to their production of numerous sulfatases [88], and their ability to adapt to such varied conditions underscores their potential role in nutrient cycling within Aiptasia, particularly in smaller anemones where rapid nutrient recycling is critical [88].

Symbiodiniaceae may function as a vehicle for Aiptasia individuals to acquire ecologically important microbial communities. Three to four weeks after inoculations, we found that 52.9%, and 29.3% respectively of the bacterial ASVs identified in the control cultures were also identified in the small and large G1 anemones respectively (Supplementary Fig. 10). We also observed a size class difference here, with 24.2% of the smaller G1 anemone’s bacteriome overlapping with the culture microbiome, whereas only 14.6% of the larger G1 anemone remained similar at the end of the experiment (Supplementary Fig. 10). In the larger anemones, beneficial bacteria including *Labrenzia* were highly relatively abundant even in the aposymbiotic colonies, but in the smaller aposymbiotic anemones it was almost completely absent. It may therefore benefit smaller Aiptasia to acquire Symbiodiniaceae early on during juvenile development to provide additional microbiota not vertically transmitted from the G0 adult colony. This may also provide an opportunity to increase the success of probiotic maintenance in Aiptasia, as introduced bacteria are lost shortly after uptake in larger anemones [89].

### Symbiodiniaceae influence on holobiont ecology is size class dependent

We found similar rates of algal uptake and proliferation across size G1 size classes (Supplementary Fig. 7). However, with microbiome modification we found that the size of G1 anemone matters, as we observed different rates of algal proliferation across treatments for each size class (Fig. 6A and B; Supplementary Fig. 7A and B) [90]. The success of proliferation, however, was inverse for the different, manipulated algae cultures for small vs large animals, with the untreated control algae infected smaller anemones slower than antifungal and antibacterial treatments (Fig. 6A and B; Supplementary Fig. 7A and B). G2 pedal lacerate production by G1 animals was highly dependent on algal symbiosis status and algal treatment, with aposymbiotic animals producing the most lacerates (Supplementary Fig. 9A). Although variable rates of algal infection and subsequent proliferation are common across cnidaria with distinct Symbiodiniaceae species [91, 92], microbial manipulation of the same algal partner supports the role of the algal microbiome as an important component for the establishment of symbiosis.

Microbiomes vary across Symbiodiniaceae (reviewed within [15]), and this community variation may contribute to the variable cnidarian proliferation rates [91, 92]. The algal microbiome may be an unexamined player in the rate of cnidarian-algal uptake, as Symbiodiniaceae derived glycans are involved in symbiosis recognition [93], but not responsible for rate of uptake [94], and onset of symbiosis is not reliant on algal photosynthesis [91]. Metabolite exchange between cnidarian symbionts plays a critical role in the ongoing stable symbioses (reviewed in [95]), including the volatilome (secondary metabolites known as biogenic volatile organic compounds) which is significantly altered in Aiptasia with the onset of symbiosis [96]. Aiptasia when in symbiosis with two different algal cultures (*B. minutum* and *Durusdinium trenchii*) presents distinct volatilome profiles that suggests an underlying mechanism to explain a metabolically inferior symbiosis with a non-native symbiont [97]. The bacteriome of these cultures was profiled and was distinct, revealing a possible relationship between the microbiome and the varied metabolite profile of different Symbiodiniaceae. Therefore, it is possible that members of the algal microbiome partially regulate onset of cnidarian symbioses although this would require further investigation.

Overall, there were no changes to Symbiodiniaceae photosynthetic efficiency, *in*- or *ex-hospite*, the growth rate of G1 anemones, or the behavior of the anemones of either size class (Supplementary Fig. 8). This is in contrast to MacVittie et al., which identified a significant reduction in anemone biomass when exposed to antibacterial compounds [33]. Although our aposymbiotic anemones grew less than our symbiotic individuals, there was no significant difference between algal treatments of either anemones size class (Supplementary Fig. 8B). Our study observed similar shifts in bacterial genera to those observed when Aiptasia were previously subjected to antibacterial compounds [36, 98]. Furthermore, we observed that the antibacterial treatment did not completely eradicate all bacteria associated with the anemones [36, 98].

We did observe a significant influence in the production of G2 pedal lacerates and their approximated relative abundance of Symbiodiniaceae across treatments, specifically in regard to hosting Symbiodiniaceae, revealing a multigenerational impact. At the end of the experiment, the G2 anemones produced by the aposymbiotic G1 animals were the most abundant, with the largest oral disc diameter (Supplementary Fig. 9B), followed by the amount of G2 offspring from G1 anemones harboring treated Symbiodiniaceae. Additionally, the G2 anemones produced by antibacterial and antifungal treated G1 anemones were visibly darker, indicating a greater algal abundance when compared to the control treatment. Taken together, hosting Symbiodiniaceae and the manipulation of the algal microbiome affects host fitness and reproduction and implies multigenerational effects that could be explored in future studies.

## Supplementary Material

2025_08_20_SI_figures_wraf189

## Data Availability

Sequences are available under the BioProject submission PRJNA1254620.
