# Supplementary material for: Manipulation of the Symbiodiniaceae microbiome confers multigenerational impacts on symbioses and reproductive ecology of its Exaiptasia diaphana host
Source: ISME J. 2025 Sep 2;19(1):wraf189. doi: 10.1093/ismejo/wraf189 (PMC12527260; doi:10.1093/ismejo/wraf189)

Supplementary Figure 1. Visualization of *Breviolum minutum* within G1 Aiptasia. a-d, Processing of epifluorescence images in ImageJ. A, Anemone viewed under fluorescence microscope with laser excitation inducing algal fluorescence. B, 2D product of a merged Z-stack video of the tentacles. C, Conversion of the color image into a greyscale image. D, ImageJ false color detection of cells for enumeration.

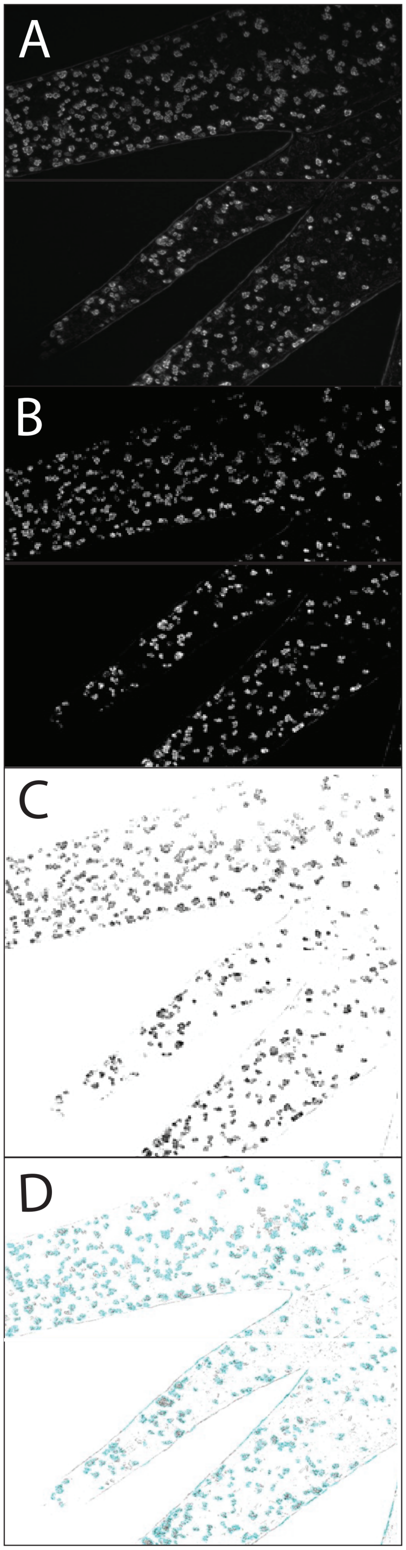

Supplementary Figure 2. Rapid light curves of experimental SSB01 (*Breviolum miniutum*) cultures. Photochemical efficiency measurements across intervals of increasing light intensities taken at the A, start and B, end of the experiment.

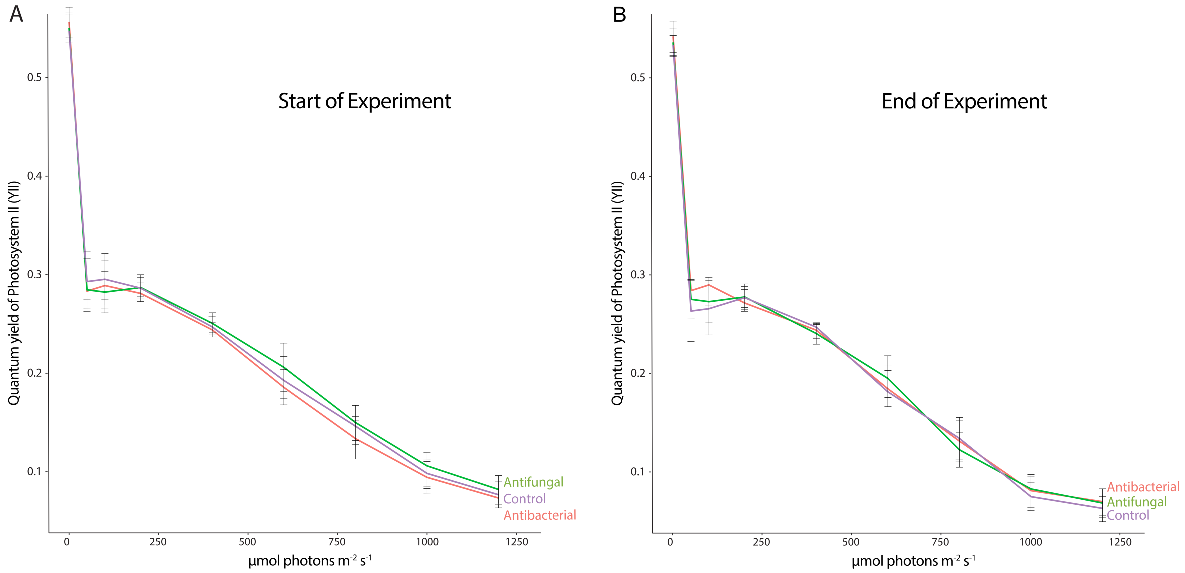

Supplementary Figure 3. Bacterial (16S rRNA gene V4 region) and fungal (18S rRNA gene V7-V8 region) community comparison between G0 (aposymbiotic) and G1 (aposymbiotic) large and small anemones. A, Significant differences observed across multiple alpha diversity indices, including crichness, abundance, and evenness, for both bacterial and fungal communities. B, Top 10 most abundant bacterial and fungal families. C, Heatmap displaying the top 7 genera of bacteria and fungi found to be most significantly different between generation and size class. Bray Curtis dissimilarity (community composition) for D, bacterial and E, fungal communities. Unique and shared F, bacteria, and G, fungi, identified across the aposymbiotic generations and size classes.

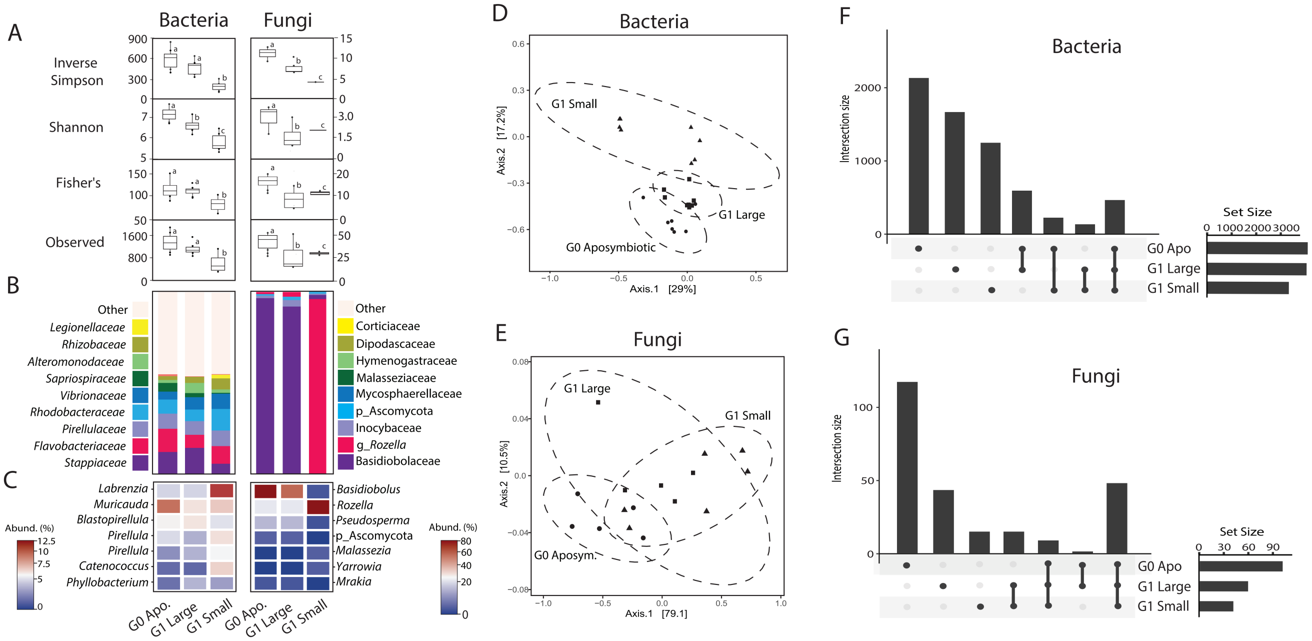

Supplementary Figure 4. Unique and shared bacteria identified across the G1 anemones in the four experimental treatments. ASVs shared between A, Small and B, Large anemones.

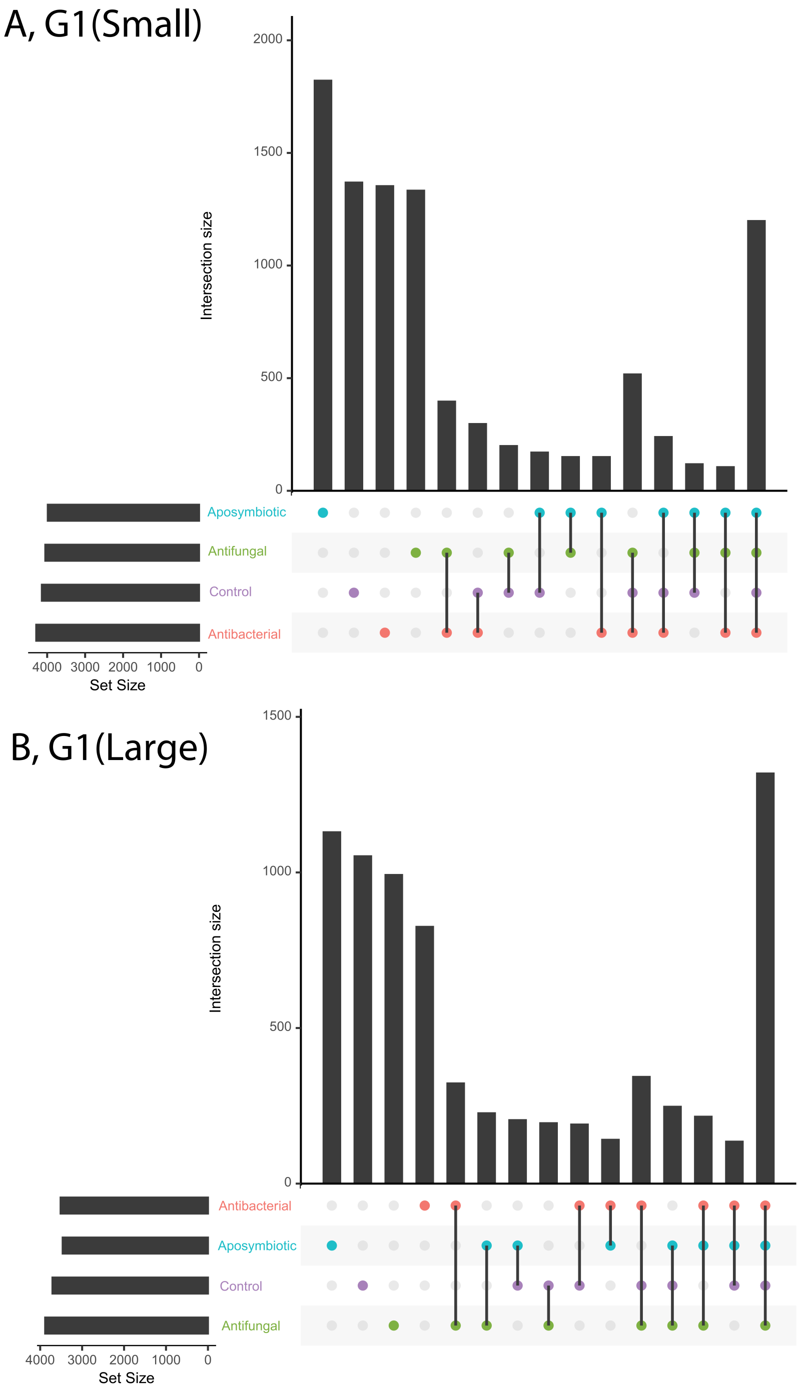

Supplementary Figure 5. Heatmap revealing bacterial detection and prevalence rates across all inoculated G1 anemones. Smaller core bacterial community present in the small anemone size class when compared to larger size class.

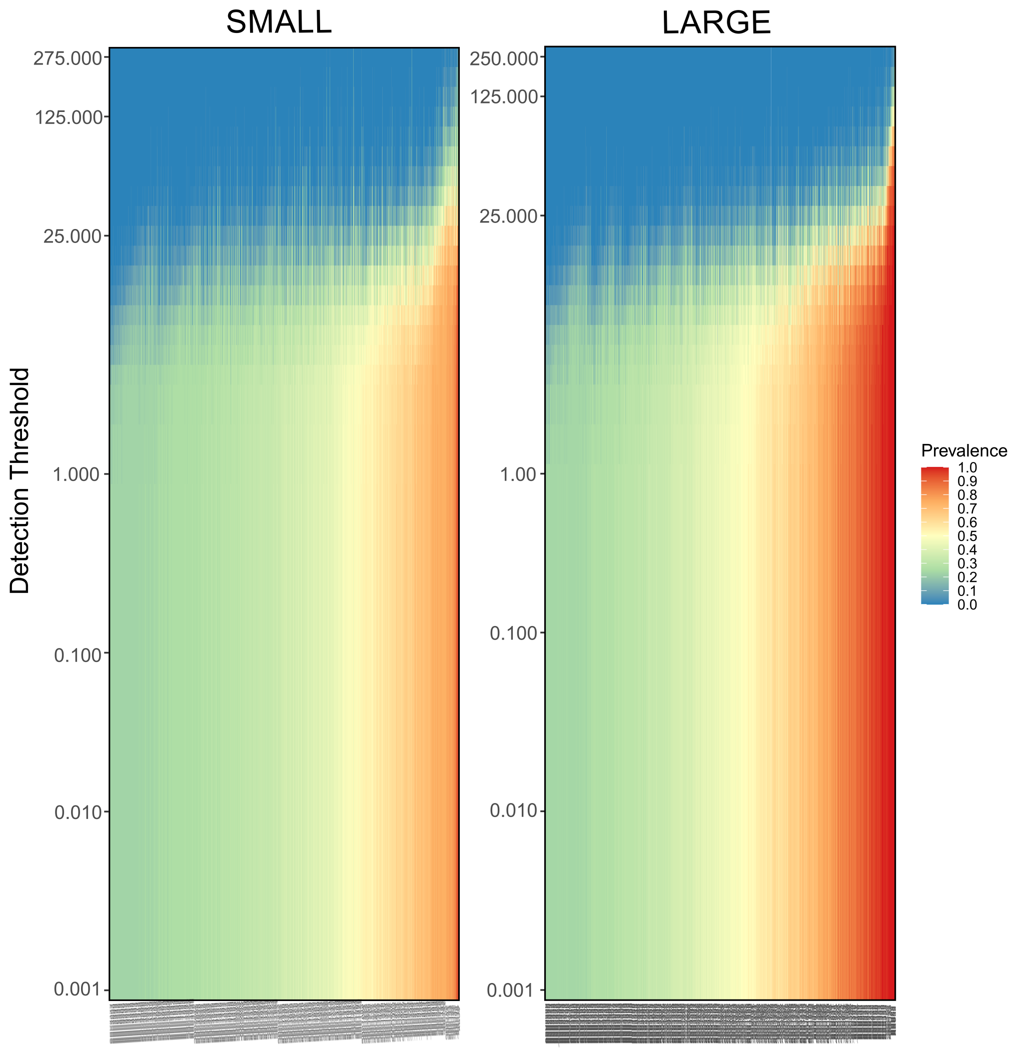

Supplementary Figure 6. Fungal (18S rRNA gene V7-V8 region) community comparison between large and small inoculated G1 anemones. A, Fungal community patterns observed across multiple alpha diversity indices for richness, abundance, and evenness for small and large anemone size classes. B, Top 10 most abundant fungal families. C, Heatmap displaying the top 7 genera of fungi found to be most significantly different between experimental treatment. Bray Curtis dissimilarity (community composition) for D, small and E, large anemones. Unique and shared fungi in F, small, and G, large anemones.

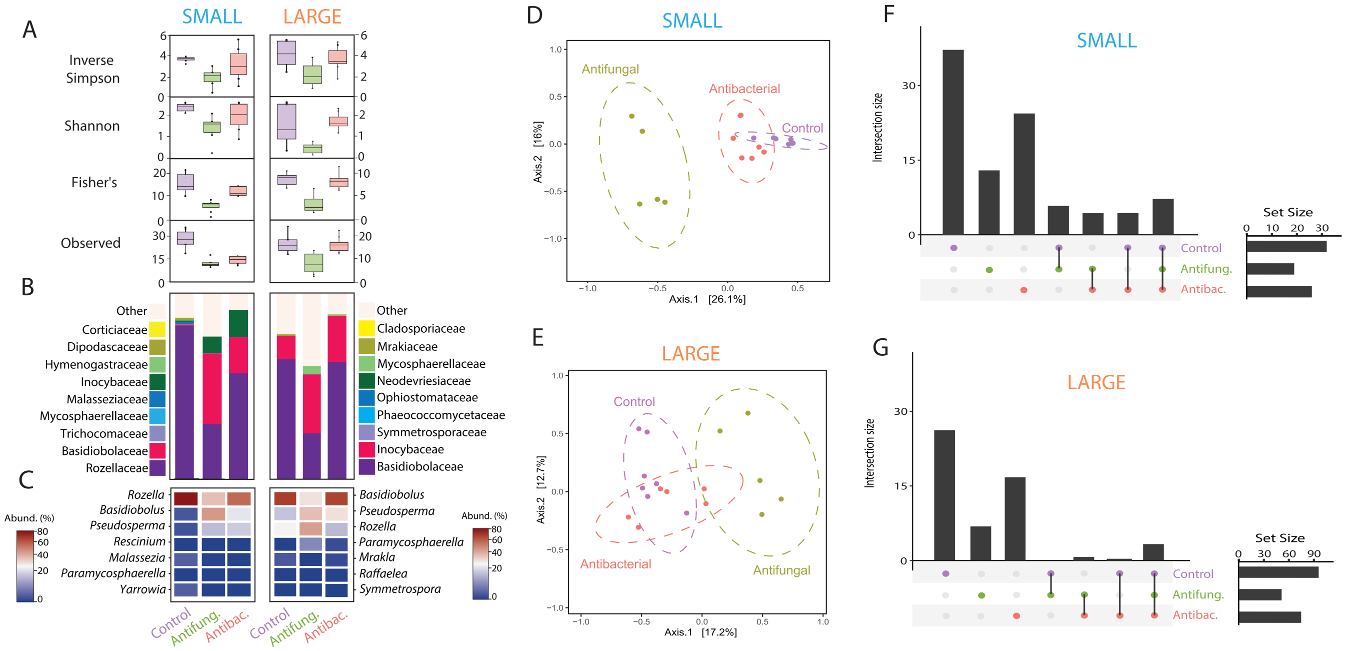

Supplementary Figure 7. Symbiodiniaceae proliferation rates within Aiptasia. Algal density changes per observation (every two days) for the A, small and B, large size class. The experiment lasted 20 days for the small size class and 26 days for the large size class.

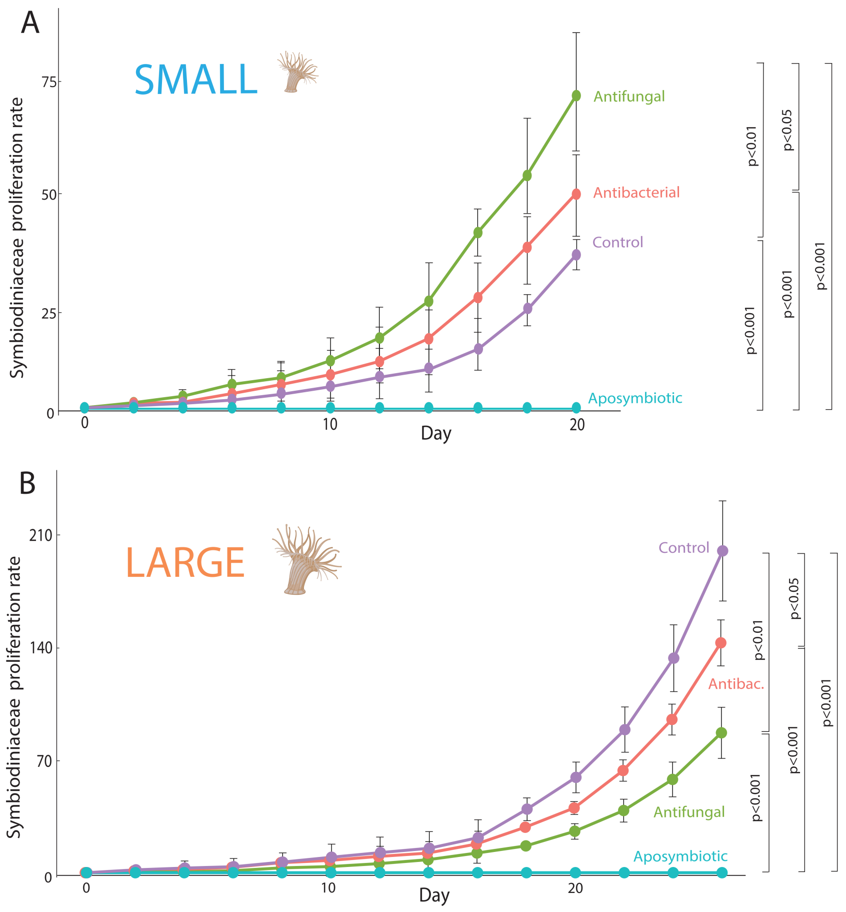

Supplementary Figure 8. G1 Host Aiptasia anemone parameters. A, Maximum photochemical potential of Photosystem II of small and large anemones measured at the end of the experiment.
B, Growth of small and large anemones based on the change in diameter of their oral disc at the end of the experiment when compared to the start. C, Behavior of tentacles across size based on visualization of tentacles either being “retracted” or ”expanded”, observed at the same time every two days throughout the experiment.

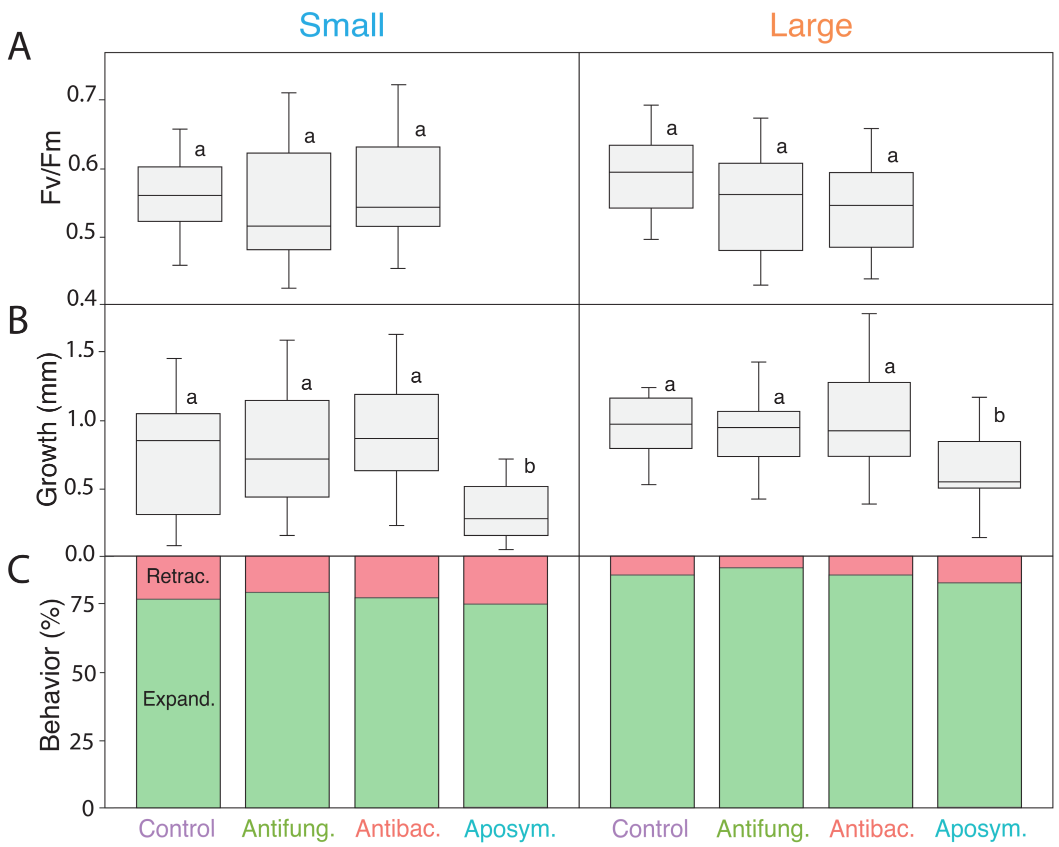

Supplementary Figure 9. G2 lacerate production density and size. A, Significantly variable mean of G2 pedal lacerates produced from all large G1 anemones in each of the four experimental treatments. B, Oral disc width of the G2 anemones, and a qualitative assessment of their color (translucent, pale yellow, light beige, dark brown) to infer algal density.

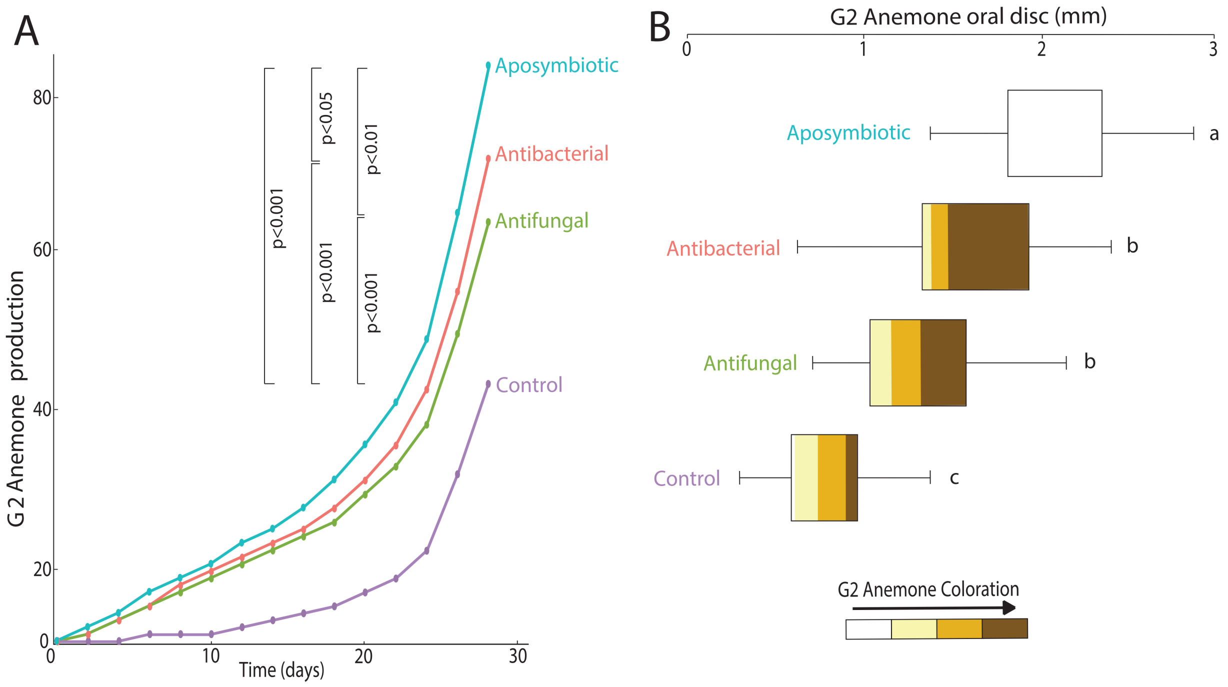

Supplementary Figure 10. Bacterial ASVs identified in the control SSB01 cultures, and G1 anemones in the untreated algal treatment. Unique and shared bacteria enumerated at the end of the experiment across the algal culture, small, and large G1 anemones.

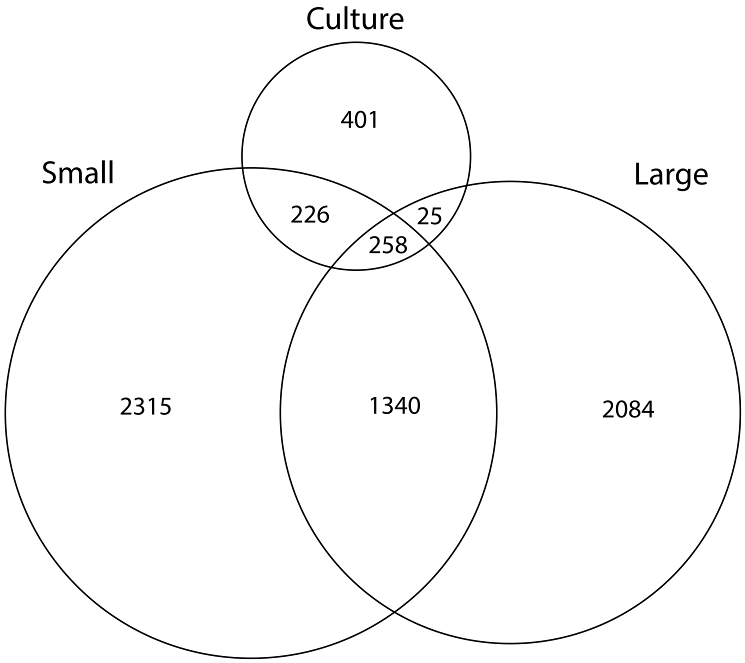

Supplement: 2025_08_20_SI_figures_wraf189 [file 2025_08_20_si_figures_wraf189.pdf]
